# Effect of Acute Ingestion of Green Tea Extract and Lemon Juice on Oxidative Stress and Lipid Profile in Pigs Fed a High-Fat Diet

**DOI:** 10.3390/antiox8060195

**Published:** 2019-06-23

**Authors:** Xi Fang, Michael Azain, Kristi Crowe-White, Jennifer Mumaw, Janet A. Grimes, Chad Schmiedt, Michele Barletta, Srujana Rayalam, Hea Jin Park

**Affiliations:** 1 Department of Foods and Nutrition, University of Georgia, Athens, GA 30602, USA; xi.fang25@uga.edu; 2 Department of Animal and Dairy Science, University of Georgia, Athens, GA 30602, USA; mazain@uga.edu; 3 Department of Human Nutrition, University of Alabama, Tuscaloosa, AL 35487, USA; kcrowe@ches.ua.edu; 4 Department of Population Health, College of Veterinary Medicine, University of Georgia, Athens, GA 30602, USA; jmumaw@uga.edu; 5 Department of Small Animal Medicine and Surgery, College of Veterinary Medicine, University of Georgia, Athens, GA 30602, USA; jgrimes@uga.edu (J.A.G.); cws@uga.edu (C.S.); 6 Department of Large Animal Medicine, College of Veterinary Medicine, University of Georgia, Athens, GA 30602, USA; barletta03@gmail.com; 7 Department of Pharmaceutical Sciences, Philadelphia College of Osteopathic Medicine, Suwanee, GA 30024, USA

**Keywords:** antioxidant enzymes, catechins, cholesterol, citrus fruits, lipid peroxidation, triacylglycerol

## Abstract

Green tea and its catechins have been shown to ameliorate high fat diet-induced oxidative stress and hyperlipidemia. However, low bioavailability of catechins limits their therapeutic potential. Lemon juice (LJ) has been suggested to enhance the bioavailability of catechins in vitro. This study investigated the antioxidative and hypolipidemic efficacy of a single dose of green tea extract (GTE) or GTE plus LJ (GTE + LJ) in high-fat diet fed pigs. Sixteen pigs ingested a single dose of GTE (190 mg/kg/day) or GTE + LJ (0.75 mL/kg/day) mixed with low-fat (LF; 5% fat) or high-fat (HF; 22% fat) diets and blood samples were collected for 24 h. Plasma catechin level peaked at two hours, and gradually returned to baseline after six hours following the intake. The addition of LJ significantly increased plasma catechin level. The diet containing GTE did not lower plasma cholesterol and triacylglycerol (TG) concentrations, superoxide dismutase (SOD) and catalase activity, or malondialdehyde concentration in 24 h in HF-fed pigs. Addition of a single dose of LJ, however, significantly decreased plasma TG level in LF groups but did not cause further changes on any other markers compared to the GTE alone. Our findings indicate limited effect of a single meal containing GTE on plasma antioxidant enzymes, lipid profile, and lipid peroxidation in pigs and no significant synergistic/additive action of adding LJ to GTE within 24 h in pigs. A study with a longer treatment period is warranted to further understand the potential role of GTE in reducing HF diet-induced oxidative stress and the possible synergistic role of LJ.

## 1. Introduction

Oxidative stress is a key component in numerous metabolic disorders and disease pathologies, such as diabetes, cardiovascular disease, and cancer [1]. Diet pattern is of great importance as a modulatory risk factor for oxidative stress in the body [2]. A high-fat (HF) diet often results in adverse metabolic outcomes where oxidative stress is increased by free radical production and an elevated inflammatory response characterized by macrophage migration and higher levels of inflammatory cytokines [3,4]. 

Green tea (*Camellia sinensis*) is a popular beverage consumed worldwide for its long history and numerous health benefits. The major polyphenolic compounds in green tea are (−)-epigallocatechin-3-gallate (EGCG), (−)-epicatechin-3-gallate (ECG), (−)-epigallocatechin (EGC), and (−)-epicatechin (EC), among which EGCG is the most abundant, accounting for more than 50% of catechin [5]. Green tea and its catechins exhibit anti-oxidative and anti-inflammatory properties making them potentially potent therapeutics for diseases where these processes are critical negative elements [6,7,8,9,10]. Green tea supplementation has been shown to have beneficial effects by reducing oxidative stress, high blood pressure, lipid absorption and glucose intolerance induced by a HF diet. 

However, findings with short-term supplementation are inconsistent. In humans, green tea supplementation for seven and 28 days decreased systolic blood pressure in obese and prehypertensive women [11] and attenuated postprandial blood glucose and insulin response in overweight men [12], respectively. Walkowiak et al., [13] also reported a decrease in lipid digestion and absorption after a single dose of green tea extract in healthy human subjects. Inconsistently, two days of green tea intervention showed no beneficial effect on glucose, insulin, and free fatty acids levels [14] and on resting metabolism [15] in healthy men and women. In addition, a single dose of green tea polyphenols did not ameliorate oxidative stress and muscle damage induced by exercise in healthy athletes [16]. These discrepancies suggested that one potential factor leading to these divergent outcomes may be related to the treatment period and emphasized necessity of elucidating acute single dose ingestion of catechins in the antioxidative efficacy of green tea. 

Furthermore, the oral bioavailability of green tea catechins is very poor with most catechins being cleared through the gastrointestinal (GI) tract before making it into circulation [17]. An enhancement in uptake and delivery of catechins would significantly improve the beneficial efficacy of green tea. Green et al. [18] demonstrated that the addition of citrus juice modulates in vitro digestive recovery of green tea catechins and lemon juice showed the maximum catechin recovery among various citrus fruits. An in vitro study [19] suggested that ascorbic acid improves catechin’s bioavailability in rats. Intriguingly, in humans, a greater consumption of citrus fruits was associated with a lower incidence of stroke in Japan [20] and a lower risk of fatal stroke in the UK [21]. Lemon has been reported to possess health-promoting activity related to cancer, cardiovascular diseases, obesity, gastrointestinal diseases, diabetes, urinary diseases, psychiatric diseases, and bone protection [22]. Lemon juice contains antioxidants such as ascorbic acid, phenols, and flavonoids and possesses antioxidant capacity [22,23]. In our current study, we hypothesize that the combination of green tea extract and lemon juice may have a synergistic effect on antioxidant activity due to the increase in catechin’s bioavailability.

Therefore, the present study aimed to investigate the antioxidative efficacy of a single dose of green tea extract (GTE), and the synergistic effect of green tea and lemon juice (GTE + LJ) in combination with low-fat (LF) or HF diet in a pig model. This animal model has proven to be superior to rodent models in studying absorption and metabolism due to similarities in gastrointestinal structures and disease progression between pigs and humans [24,25].

## 2. Materials and Methods 

### 2.1. Animals and Study Design

**Figure 1:**
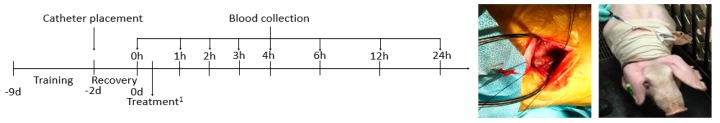
Study design. ^1^ Pigs were provided with either GTE (190 mg/kg/treatment) or GTE + L (190 mg/kg/treatment GTE + 0.75 mL/kg lemon juice/treatment) mixed in LF (5% fat diet) or HF diet (22% fat diet). After blood collection at the 0-time point, samples were collected at 1, 2, 3, 4, 6, 12, and 24 h post-treatment. GTE: green tea extract; LF: low-fat; HF: high-fat.

The cross-bred commercial line of healthy male and female pigs (*n* = 16) was obtained from the University of Georgia swine farm at the age of 15 weeks with a body weight of 37–49 kg. After two weeks of acclimation in a temperature controlled facility, they were trained to consume a meal within 30 min for one week. A catheter was surgically implanted into the jugular vein of each pig and a 2-day recovery period was allowed prior to the experiment. Pigs were randomly assigned to four treatment groups: low-fat diet with green tea extract (LF + GTE) (*n* = 5), high-fat diet with green tea (HF + GTE) (*n* = 4), low-fat diet with green tea extract and lemon juice (LF + GTE + LJ) (*n* = 3), and high-fat diet with green tea extract and lemon juice (HF + GTE + LJ) (*n* = 4). Blood samples were collected before treatment as a baseline. Each animal at baseline measurement served as its own control. Blood samples were obtained through the jugular catheter at baseline (pretreatment), 1, 2, 3, 4, 6, 12, and 24 h post-consumption of experimental diets (Figure 1). This study was conducted in accordance with the University of Georgia Institutional Animal Care and Use Committee guidelines (project code: A2016 06-011-Y3-A5).

### 2.2. Treatments 

All animals were maintained on a grower diet primarily composed of corn with a total of 3400 kcal/kg to meet normal metabolism and growth requirement according to NRC, 2012 [26]. Pigs in the LF group consumed the baseline diet throughout the study (5% fat diet). The HF diet was prepared by mixing the base diet with 20% lard (wt/wt) (designated as the 22% fat diet). The dose of GTE was selected based on literature reporting beneficial health effects in human subjects [27,28,29]. In order to accurately translate data from pigs to humans, the doses of GTE were adjusted using standard FDA conversions between animal and human equivalent dose [30], which resulted in 1.3 g/pig/day of GTE (90M-B Sunphenon, The Food Grade, non GMO powdered GTE, Taiyo International, Inc., Minneapolis, MN, USA), containing > 75% (wt/wt) total catechins including > 40% and < 10% Caffeine EGCG as verified by HPLC. This is equivalent to 5 cups/day of human consumption which has been shown to decrease cardiovascular disease mortality in the Japanese population [31]. The dose of lemon juice (ReaLemon, Dr. Pepper Snapple Group) was selected based on the FDA reports of lemon juices used as ingredients [32]. The LF or HF diet were mixed with GTE (190 mg/kg/treatment) or GTE + LJ (190 mg/kg/treatment GTE + 0.75 mL/kg lemon juice/treatment).

### 2.3. Plasma Catechin Analysis 

Blood was collected in EDTA-coated tubes, and plasma was separated by centrifuging blood at 2000× *g* for 10 min at 20 °C. Plasma EGCG and EGC levels were analyzed using a previously validated ultra-high performance liquid chromatography (UPLC) method for analysis of tea catechins [33]. The Acquity UPLC system was interfaced with a photodiode array detector and a quaternary solvent manager (Waters Corporation, Milford, MA, USA) and the instrument was fitted with an Acquity UPLC HSS T3 column (100 mm × 2.1 mm, 1.8 um) protected with a 0.2 um in-line filter. Reference standards included EGCG (97%) and EGC (95%) purchased from Sigma (St. Louis, MO, USA). Standard stock solutions were prepared in 3% acetonitrile and further diluted with 3% acetonitrile to obtain different concentration levels for preparation of standard curves.

### 2.4. Plasma Cholesterol and Triglyceride

To measure antioxidant defense after acute administration of test diets, plasma superoxide dismutase (SOD) and catalase were analyzed colorimetrically using superoxide dismutase assay kit (Cayman, Ann Arbor, MI, USA) and catalase assay kit (Cayman, Ann Arbor, MI, USA) according to the manufacturer’s instructions.

### 2.5. Liver Function Tests

To determine whether the dose we chose causes any hepatotoxicity, liver alanine aminotransferase (ALT), aspartate aminotransferase (AST), total bilirubin, and gamma-glutamyl transferase (GGT) were measured using a clinical chemistry analyzer.

### 2.6. Plasma Lipid Peroxidation

With the aim of exploring the potential protective effect of GTE and GTE + LJ on oxidative stress, the plasma level of malondialdehyde was analyzed spectrophotometrically as an indicator of lipid peroxidation using thiobarbituric acid reactive substances (TBARS) assay kit (Cayman, Ann Arbor, MI, USA) according to the manufacturer’s instruction.

### 2.7. Activities of Plasma Antioxidant Enzymes

To measure antioxidant defense after acute administration of test diets, plasma superoxide dismutase (SOD) and catalase enzyme activities were analyzed colorimetrically using superoxide dismutase assay kit (Cayman, Ann Arbor, MI, USA) and catalase assay kit (Cayman, Ann Arbor, MI, USA) according to the manufacturer’s instructions.

### 2.8. Statistical Analysis

Data expressed as mean ± S.E. were analyzed using Graph Pad Prism (Version 7.00; GraphPad Software, Inc.; San Diego, CA, USA). One-way analysis of variance (ANOVA) with Tukey Honestly Significant Difference (HSD) post-hoc test and paired t-test were applied to evaluate mean differences between groups and between time points, respectively.

## 3. Results

### 3.1. Plasma Catechin Level

**Figure 2:**
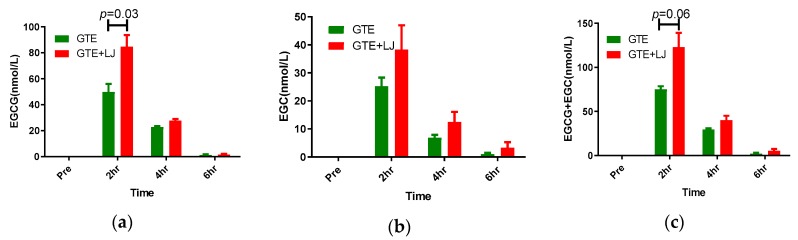
Plasma EGCG (**a**), EGC (**b**), and EGCG + EGC (**c**) levels after one-time ingestion of GTE and GTE + LJ with LF diet in pigs (*n* = 3–4). EGCG: (−)-epigallocatechin-3-gallate; ECG: (−)-epicatechin-3-gallate.

In order to determine the level of catechin in the systemic circulation, plasma EGCG and EGC were measured at 2, 4, and 6 h after the acute intake of LF diet with GTE or GTE + LJ in pigs. In both GTE and GTE + LJ groups, plasma EGCG and EGC concentration increased dramatically upon ingestion and peaked at two hours after the intake (EGCG: 49.77 ± 6.26, 84.66 ± 8.97 nmol/L; EGC: 25.28 ± 3.07, 38.36 ± 8.65 nmol/L, EGCG + EGC: 75.05 ± 6.20, 123.01 ± 32.59 nmol/L in GTE and GTE + LJ, respectively). The peak concentration of EGCG in GTE + LJ group was 1.7-fold higher than GTE group (*p* = 0.03). Similarly, the peak circulating levels of EGCG + EGC in GTE + LJ was 1.64-fold higher than GTE group (*p* = 0.06). Plasma catechin concentration went back to baseline (below 2 nmol/L) six hours following the treatment in both GTE and GTE + LJ groups (Figure 2).

### 3.2. Plasma Lipid Profile

**Figure 3:**
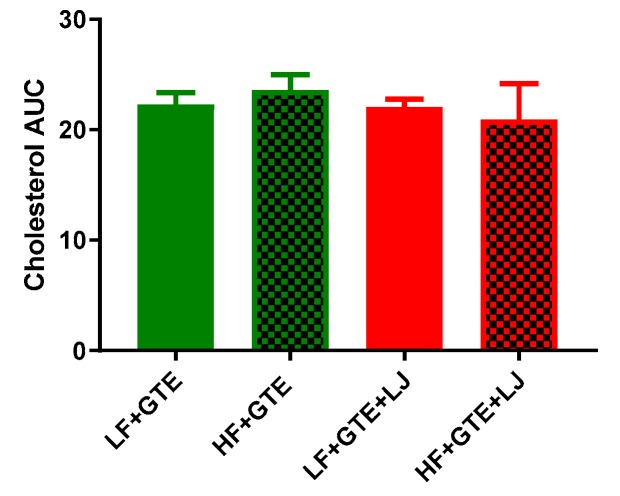
Area under the curve (AUC) of plasma cholesterol concentration after a single dose of LF, HF, LF + GTE, HF + GTE, LF + GTE + LJ, and HF + GTE + LJ in pigs. Data present means ± SEM; *n* = 3–5 pigs per group. Significance of differences between groups is shown.

Green tea and its catechins have been documented for their hypolipidemic activity. Plasma cholesterol and TG were measured after a single-dose of GTE or GTE + LJ with LF or HF diet in pigs. In our study, different fat content did not alter postprandial cholesterol and TG levels comparing LF + GTE and HF + GTE groups. In LF diet-fed groups, postprandial cholesterol level decreased up to 7% at four hours which then returned to baseline, while a combination of GTE + LJ did not further influence plasma cholesterol within 24 h (Table 1 and Figure 3). In HF diet-fed groups, cholesterol was not altered by acute intake of GTE and combination of GTE + LJ.

**Figure 4:**
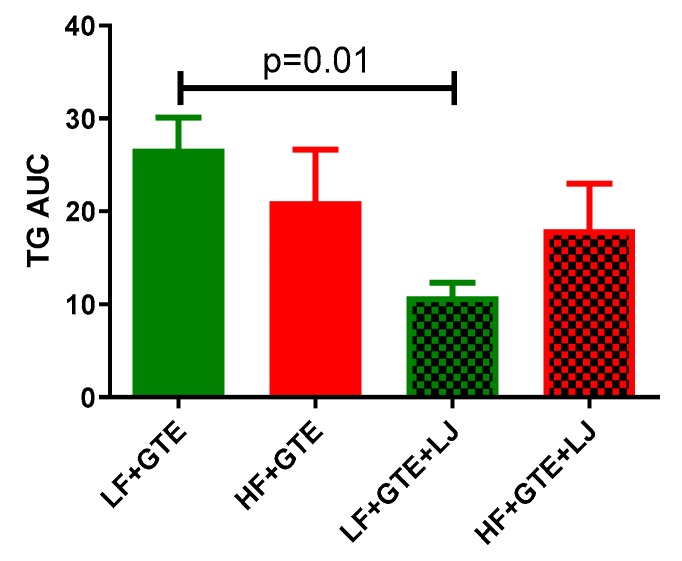
Area under the curve (AUC) of plasma triglycerides (TG) concentration after a single dose of LF, HF, LF + GTE, HF + GTE, LF + GTE + LJ, and HF + GTE + LJ in pigs. Data present means ± SEM; *n* = 3–5 pigs per group. Significance of differences between groups is shown.

In LF + GTE group, plasma TG concentration was decreased up to 20% at one hour and six hours timepoint compared to preprandial level which returned to baseline levels within 24 h (Table 2). In LF + GTE + LJ group, plasma TG concentration dropped up to 53% 2–12 h following the intake compared to preprandial level (Table 2). Plasma incremental TG AUC was significantly lower in LF + GTE + LJ group compared to LF + GTE group (*p* = 0.01) (Figure 4). In HF fed-groups, postprandial TG concentration significantly decreased at 4, 6, 12 h and 3, 4, 6 h compared to baseline level in HF + GTE group and HF + GTE + LJ groups, respectively (Table 2). However, between HF + GTE and HF + GTE + LJ groups, there was no significant difference in the TG concentration.

### 3.3. Liver Function Tests

The doses of GTE and LJ tested in the current study did not cause liver toxicity. No significant changes were noticed in ALT activity, AST activity, total bilirubin, and GGT levels (data not shown).

### 3.4. Plasma Lipid Peroxidation

**Figure 5:**
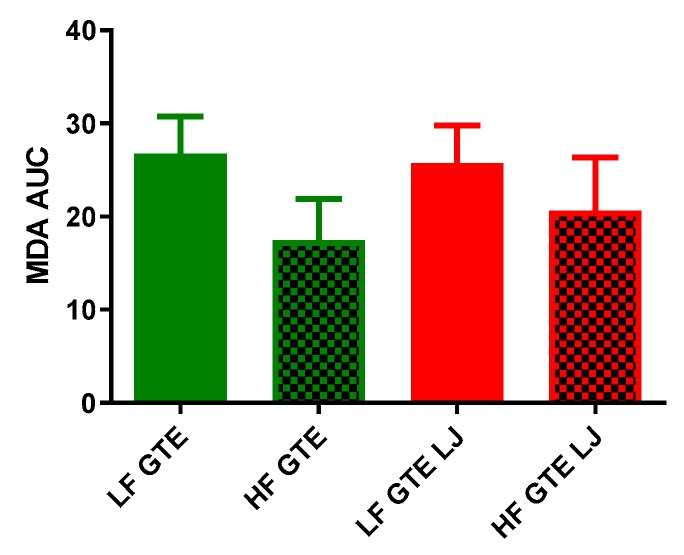
Area under the curve (AUC) of plasma malondialdehyde (MDA) concentration after a single dose of LF, HF, LF + GTE, HF + GTE, LF + GTE + LJ, and HF + GTE + LJ in pigs. Data present means ± SEM; *n* = 3–5 pigs per group. Significance of differences between groups is shown.

HF diet has been reported to induce an elevation in plasma lipid content and potentiate systemic oxidative stress [34,35], while chronic treatment with catechins has been reported to normalize elevated lipid peroxidation induced by HF diet in rats [36]. Thus, we measured plasma malondialdehyde (MDA) concentration to investigate whether acute ingestion of GTE or the combination of GTE + LJ would be sufficient to reduce lipid peroxidation in HF-fed pigs. As shown in Table 3 and Figure 5, a single dose of HF diet had no significant effect on postprandial plasma MDA level comparing LF + GTE and HF + GTE groups. In LF diet-fed groups, MDA concentration did not change following GTE or GTE + LJ ingestion. In HF diet-fed groups, MDA level tended to decrease at one and three hours by 16% postprandial in HF + GTE group, which went back to baseline after four hours. Addition of LJ with GTE also decreased MDA up to 45% within the first two hours of ingestion but then recovered to baseline level after four hours (Table 3).

### 3.5. Activities of Plasma Antioxidant Enzymes

**Figure 6:**
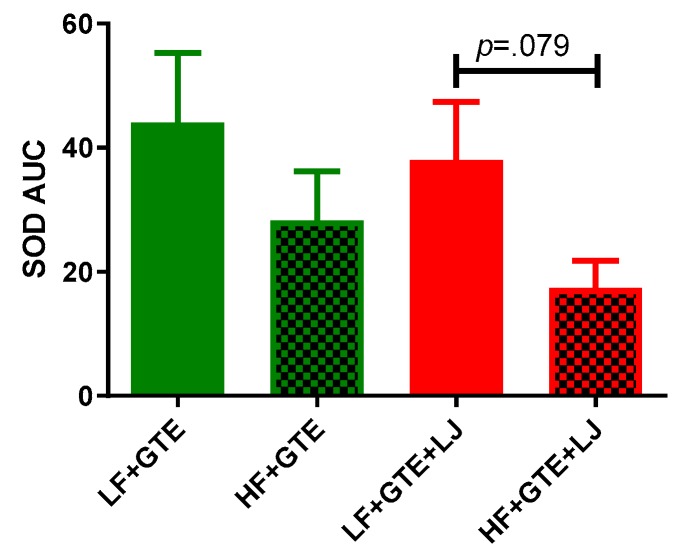
Area under the curve (AUC) of plasma superoxide dismutase (SOD) activity after a single dose of LF, HF, LF + GTE, HF + GTE, LF + GTE + LJ, and HF + GTE + LJ in pigs. Data present means ± SEM; *n* = 3–5 pigs per group. Significance of differences between groups is shown.

**Figure 7:**
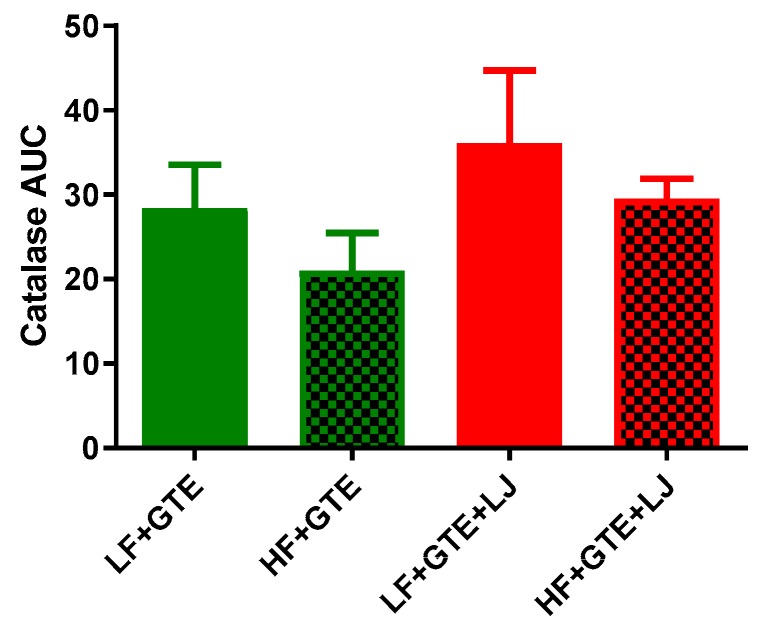
Area under the curve (AUC) of plasma catalase activity after a single dose of LF, HF, LF + GTE, HF + GTE, LF + GTE + LJ, and HF + GTE + LJ in pigs. Data present means ± SEM; *n* = 3–5 pigs per group. Significance of differences between groups is shown.

Activities of plasma SOD and catalase were measured to indicate antioxidant defense in the HF diet-fed pigs after acute exposure to either GTE or GTE + LJ. As shown in Table 4 and Figure 6, different fat content did not exert a significant effect on postprandial plasma SOD activity comparing LF + GTE and HF + GTE groups. However, Plasma incremental SOD AUC showed a trend of decrease in HF + GTE + LJ compared to LF + GTE + LJ group. In LF + GTE group, the plasma SOD activity increased 68% from one hour following the meal, returned to the baseline level at six hours, and again increased up to 139% at 24 h following intake comparing to preprandial level. The GTE + LJ group showed a similar trend as GTE alone but no significance was observed which may be due to the small number of animals in the group. In HF diet-fed pigs, no change was observed in the activity of SOD. The addition of LJ did not exert an apparent effect on plasma SOD activity comparing HF + GTE and HF + GTE + LJ groups (Table 4 and Figure 6). As shown in Table 5 and Figure 7, when GTE was consumed with HF diet, postprandial catalase activities tended to be lower compared to LF + GTE group. In LF diet-fed pigs, postprandial plasma catalase activities tended to be higher regardless of fat content comparing LF + GTE + LJ to LF + GTE and HF + GTE + LJ to HF + GTE groups, however, none of these reached the statistical significance cut-off point. Taken together, our data revealed that GTE had a limited influence on plasma SOD and catalase activity, and LJ did not modulate this effect within 24 h.

### 3.6. Correlation between Plasma Antioxidant Enzymes and Triglycerides 

**Figure 8:**
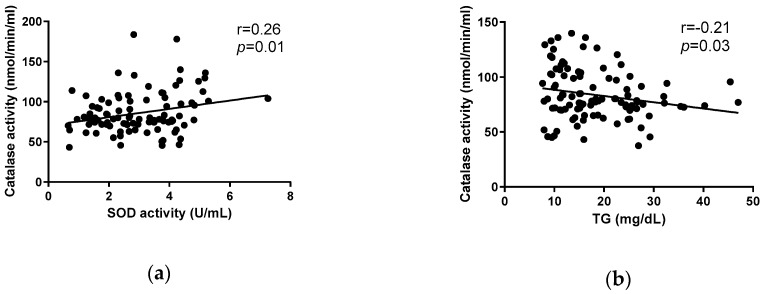
Correlation of plasma superoxide dismutase activity and catalase activity (**a**) and triglycerides (TG) concentration and catalase activity (**b**) after a single dose of LF, HF, LF + GTE, HF + GTE, LF + GTE + LJ, and HF + GTE + LJ in pigs. Data present means ± SEM; *n* = 3–5 pigs per group. Pearson coefficient (r) and *p*-value is shown.

Pearson correlation analysis revealed a positive correlation between plasma catalase activity and plasma SOD activity (*r* = 0.26, *p* = 0.01), and a negative correlation between plasma catalase activity and plasma TG level (*r* = −0.21, *p* = 0.03) (Figure 8).

## 4. Discussion

Green tea and its catechins possess therapeutic potential for chronic diseases by mitigating oxidative stress and hyperlipidemia. The low bioavailability of catechins limits their therapeutic potential, and there are numerous studies attempting to overcome this challenge by enhancing their bioavailability including strategies such as encapsulation, nanoparticle delivery or combining with other foods. One of the safest and most novel approaches is the combination of citrus fruits with green tea, which has been found to increase the bioavailability of green tea catechin in an in vitro digestion model [18]. In accordance with findings reported by Huang et al., and Lee et al., we found plasma concentration of catechin peaked at two hours upon single dose ingestion and gradually decreased to baseline between 6–8 h after oral consumption both in rodents and humans [37,38]. Moreover, the present study reported a significant increase in circulating catechin level when GTE was ingested in combination with LJ, which is in agreement with Peters et al.,’s finding that the addition of ascorbic acid and sucrose significantly increased plasma EGCG and EGC concentration within two hours after the ingestion in rats [19]. We hypothesize that several factors might have contributed to the observed improvement in blood catechin levels with GTE + LJ compared to LJ alone. Polyphenol-rich tea drinks are susceptible to form precipitates referred to as tea cream, caused by the interactions of catechin, proteins, and other polyphenols [39]. The precipitation is formed more readily under acidic conditions like gastric environment [40]. Gallated catechins including EGCG, (−)-gallocatechin-3-gallat (GCG), ECG, and (−)-catechin-3-gallate (CG) were found to have higher tendency to form cream compared to other catechins [41]. The ascorbic acid in LJ was shown to improve the circulating catechin level likely by improving the stabilization of these labile catechins in the gastrointestinal tract [18]. Additionally, ascorbic acid was found to increase the stability of catechin and its derivatives especially for EGC and EGCG in vitro [42], suggesting a possible explanation for the increased plasma catechin levels in GTE + LJ group.

In accordance with previous research, postprandial cholesterol did not change significantly after the intervention meal [43,44]. In addition, in this study, we did not observe a significant difference in plasma TG levels between LF + GTE and HF + GTE groups, suggesting that the one-time increase in fat content did not impact plasma TG when HF was consumed with GTE. This is likely due to the hypolipidemic effect of green tea. In normal human subjects, postprandial plasma TG increases in response to a HF meal around two hours [45]. In pigs, postprandial TG decreased one hour after the meal and then increased after two hours [46]. GTE significantly decreased plasma and hepatic TG 6 weeks after feeding in mice [47]. Another study found that four-day EGCG supplementation decreased liver and plasma TG level compared to the HF diet-fed controls in mice [48]. Additionally, green tea catechin has also been reported to decrease postprandial plasma TG in human subjects [49]. In this study, postprandial TG levels of all treatment groups were decreased within six hours after the meal, which was further decreased by the addition of LJ under normal conditions in a pig model. These findings may suggest that GTE, especially when consumed with LJ, prevented the increase of plasma TG level after a meal. 

We found that when combined with GTE, higher dietary fat content resulted in decreased plasma MDA concentration in the HF + GTE group compared to the LF + GTE group. Interestingly, previous findings report that chronic or repeated single HF diet increases the plasma MDA concentration [50,51]. Multiple factors can be attributed to different biological reactions to a HF meal including, macronutrient composition, different types of fat, lifestyles, and bioactive food compounds within a food matrix. Montes-Nieto et al., found that postprandial MDA increased significantly compared to the preprandial level after ingestion of glucose; however, this was not seen when ingesting a lipid diet wherein the postprandial circulating TBARS remained unchanged throughout the trial compared to baseline level following the ingestion of poly-unsaturated triglycerides nutrition supplement in young adults [52]. In another study that investigated the postprandial antioxidant defenses in physically active and inactive men, TBARS in physically active men showed a decreasing trend compared to baseline level after a HF meal [53]. In this study, the decrease of MDA concentration observed in HF + GTE diet compared to LF + GTE group may be a compensating effect due to the activation of the antioxidant effect of GTE when combined with HF diet, suggesting GTE alone, or the combination of GTE and LJ was able to suppress the elevation of plasma MDA seen by other studies when induced by HF diet [54,55].

SOD and catalase constitute two major enzymatic antioxidant defenses against free radical damage in biological system. Reactive oxygen species (ROS) are removed through dismutation of free radicals into H_2_O_2_ catalyzed by SOD, which is further converted into H_2_O by catalase [56]. In this study, we found that HF content seems to decrease plasma SOD activities comparing HF + GTE and LF + GTE, however, the overall postprandial SOD activity was increased compared to baseline in all treatment groups. Indeed, a HF diet was found to compromise the antioxidant defense system. The postprandial blood SOD activity showed a decreasing trend after a HF meal within 2–4 h compared to baseline level in exercise-trained healthy subjects [57,58]. However, it should be noted that the subjects in this study consumed a HF meal with potent bioactive compounds, which might in part explain why SOD activity was elevated in all groups. A study showed that postprandial SOD activity responds differently to different diets in subjects. After a meat meal, the plasma SOD remained at a similar level at 2–4 h following ingestion, however, after a vegan meal, the plasma SOD activity increased after 2 h of intake [59]. In addition, it was found that the postprandial plasma SOD activity increased by 3 fold four hours after a plant-based Mediterranean meal in human subjects [60]. These indicate that the increased SOD activity seen in this study might be due to the antioxidant effect of GTE regardless of fat content.

Following a HF meal, the blood catalase level decreased as a reaction to elevated oxidative stress 2–4 h postprandial in healthy exercise-trained subjects [57,58]. Antioxidant enzymes including SOD and catalase were activated after a plant-based Mediterranean meal in human subjects [60]. Long-term consumption of green tea was able to upregulate SOD and catalase enzyme activities, as was previously found in fruit flies [61]. In this study, we found that the postprandial catalase activity following the meal was elevated compared to baseline level regardless of fat content in both LF + GTE and HF + GTE groups. Especially when consumed with LJ, plasma catalase activities tended to increase further. This finding might be due to the antioxidant effect of GTE and the potential synergistic effect of GTE and LJ. However, the acute intake of GTE and LJ did not exert statistically significant antioxidant effects on pigs which can be ascribed to the short exposure time. 

In agreement with other findings, plasma SOD and catalase enzyme activities, as first-line defense antioxidants, were positively correlated in this study [62,63,64]. Additionally, plasma catalase activity was weakly correlated with plasma TG level, which is in accordance with previous findings [65].

The current study demonstrates that a single dose of GTE did not alter oxidative stress markers or lipid profile in 24 h and a single dose of lemon juice did not cause a significant improvement in GTE function in the same experimental period in a healthy pig model. However, LJ significantly increased the circulating level of green tea catechin following one-time consumption. This study used a pig model to provide insight into the potential acute antioxidative efficacy of green tea following single dose treatment, and a possibility of synergism of food components to enhance its bioavailability. The doses of GTE and LJ tested in the current study did not cause liver toxicity measured by ALT and AST activity, total bilirubin, and GGT levels (data not shown). Although acute exposure to HF diet is hypothesized to induce oxidative stress and stress-induced lipogenesis in liver, recent studies in rodent models of acute exposure to HD diet suggest that stress-induced upregulation in lipogenic markers occurs at a time point beyond 72 h [66] It is possible that the protective effects of GTE and GTE + LJ on HF-induced hepatic lipogenesis and or injury may be apparent after 72 h. Despite the reports on the hepatotoxic side effects of green tea, a recent systematic review of randomized clinical trials suggests that liver-related adverse events are rare with the intake of GTE [67].

Therefore, a future study using higher doses of GTE or LJ is suggested to investigate the reduction in oxidative stress and hyperlipidemia in HF-fed pigs. In addition, longer duration of treatments with GTE and LJ may also lead to more beneficial results.

## 5. Conclusions

Our findings revealed that a single dose of GTE had a limited effect on plasma antioxidant enzymes, lipid profile, and lipid peroxidation in HF-fed pigs in 24 h. A single dose of LJ combined with GTE did not further influence the action of GTE during the experimental period. Further study with a longer treatment period and larger sample size are warranted to elucidate the potential improved antioxidant efficacy of GTE when combined with LJ.

## Figures and Tables

**Table 1:** Plasma cholesterol (mg/dL) concentrations after a single dose of LF, HF, LF + GTE, HF + GTE, LF + GTE + LJ, and HF + GTE + LJ in pigs.

Hours Post Treatment	LF + GTE (*n* = 5)	HF + GTE (*n* = 4)	LF + GTE + LJ (*n* = 3)	HF + GTE + LJ (*n* = 4)
Pre	1.00	1.00	1.00	1.00
1	1.01 ± 0.05	1.10 ± 0.02 *	0.92 ± 0.08	0.98 ± 0.06
2	0.94 ± 0.04	0.98 ± 0.04	0.86 ± 0.05	0.99 ± 0.05
3	0.97 ± 0.02	0.92 ± 0.06	0.86 ± 0.06	0.99 ± 0.07
4	0.93 ± 0.02 *	0.95 ± 0.06	0.81 ± 0.11	0.95 ± 0.02
6	0.92 ± 0.04	1.06 ± 0.06	0.89 ± 0.11	0.77 ± 0.08 *
12	0.87 ± 0.07	0.78 ± 0.09	0.94 ± 0.03	1.16 ± 0.07
24	1.02 ± 0.05	1.01 ± 0.02	0.93 ± 0.04	1.11 ± 0.05

Abbreviation: LF: low-fat, HF: high-fat, GTE: green tea extract, LJ: lemon juice. Data were calculated as the percentage to the baseline level. * represents a significant difference (*p* < 0.05) compared to zero hours within the group.

**Table 2:** Plasma triglycerides (mg/dL) concentrations after a single dose of LF, HF, LF + GTE, HF + GTE, LF + GTE + LJ, and HF + GTE + LJ in pigs.

Hours Post Treatment	LF + GTE (*n* = 5)	HF + GTE (*n* = 4)	LF + GTE + LJ (*n* = 3)	HF + GTE + LJ (*n* = 4)
Pre	1.00	1.00	1.00	1.00
1	0.80 ± 0.09 *	0.99 ± 0.30	0.73 ± 0.13	0.96 ± 0.10
2	0.99 ± 0.11	0.73 ± 0.14	0.57 ± 0.07 *	1.01 ± 0.15
3	1.19 ± 0.12	0.74 ± 0.13	0.54 ± 0.15 *	0.85 ± 0.04 *
4	0.74 ± 0.22	0.60 ± 0.12 *	0.53 ± 0.17	0.68 ± 0.05 *
6	0.84 ± 0.07 *	0.56 ± 0.07 *	0.47 ± 0.04 *	0.54 ± 0.17 *
12	1.28 ± 0.24	1.68 ± 0.10 *	0.55 ± 0.08 *	1.42 ± 0.00
24	1.21 ± 0.24	0.94 ± 0.26	0.59 ± 0.07	1.03 ± 0.25

Abbreviation: LF: low-fat, HF: high-fat, GTE: green tea extract, LJ: lemon juice. Data were calculated as the percentage to the baseline level. * represents a significant difference (*p* < 0.05) compared to zero hours within the group.

**Table 3:** Plasma MDA concentrations (µM) after a single dose of LF, HF, LF + GTE, HF + GTE, LF + GTE + LJ, and HF + GTE + LJ in pigs.

Hours Post Treatment	LF + GTE (*n* = 5)	HF + GTE (*n* = 4)	LF + GTE + LJ (*n* = 3)	HF + GTE + LJ (*n* = 4)
Pre	1.00	1.00	1.00	1.00
1	1.12 ± 0.16	0.79 ± 0.02 *	1.08 ± 0.13	0.60 ± 0.08 *
2	1.10 ± 0.16	0.96 ± 0.14	0.93 ± 0.08	0.55 ± 0.15 *
3	0.81 ± 0.21	0.84 ± 0.05 *	1.09 ± 0.18	0.80 ± 0.15
4	1.05 ± 0.14	0.91 ± 0.11	0.79 ± 0.00	1.17 ± 0.35
6	1.30 ± 0.22	0.85 ± 0.13	0.87 ± 0.08	0.99 ± 0.23
12	1.08 ± 0.17	0.91 ± 0.06	0.95 ± 0.19	0.89 ± 0.34
24	1.10 ± 0.20	0.88 ± 0.18	1.47 ± 0.33	0.99 ± 0.15

Abbreviation: MDA: malondialdehyde, LF: low-fat, HF: high-fat, GTE: green tea extract, LJ: lemon juice. Data were calculated as the percentage to the baseline level. * represents a significant difference (*p* < 0.05) compared to zero hours within the group.

**Table 4:** Activity of plasma SOD (U/mL) after a single dose of LF, HF, LF + GTE, HF + GTE, LF + GTE + LJ, and HF + GTE + LJ in pigs.

Hours Post Treatment	LF + GTE (*n* = 5)	HF + GTE (*n* = 4)	LF + GTE + LJ (*n* = 3)	HF + GTE + LJ (*n* = 4)
Pre	1.00	1.00	1.00	1.00
1	1.68 ± 0.18 *	1.06 ± 0.36	1.11 ± 0.29	1.15 ± 0.27
2	1.95 ± 0.63	1.14 ± 0.29	1.11 ± 0.44	1.33 ± 0.33
3	1.86 ± 0.46	1.39 ± 0.44	1.51 ± 0.33	1.45 ± 0.49
4	1.97 ± 0.58	1.41 ± 0.37	1.80 ± 1.07	1.30 ± 0.24
6	1.05 ± 0.35	1.83 ± 0.29	0.56 ± 0.35	0.81 ± 0.18
12	2.25 ± 0.51 *	1.32 ± 0.48	1.92 ± 0.43	0.89 ± 0.18
24	2.39 ± 0.52 *	1.32 ± 0.34	1.93 ± 0.61	1.28 ± 0.06

Abbreviation: SOD: superoxide dismutase, LF: low-fat, HF: high-fat, GTE: green tea extract, LJ: lemon juice. Data were calculated as the percentage to the baseline level. * represents a significant difference (*p* < 0.05) compared to zero hours within the group.

**Table 5:** Activity of plasma catalase (nmol/min/mL) after a single dose of LF, HF, LF + GTE, HF + GTE, LF + GTE + LJ, and HF + GTE + LJ in pigs.

Hours Post Treatment	LF + GTE (*n* = 5)	HF + GTE (*n* = 4)	LF + GTE + LJ (*n* = 3)	HF + GTE + LJ (*n* = 3)
Pre	1.00	1.00	1.00	1.00
1	1.08 ± 0.11	1.05 ± 0.13	1.23 ± 0.21	0.93 ± 0.06
2	0.99 ± 0.17	1.06 ± 0.20	1.43 ± 0.69	0.96 ± 0.19
3	0.75 ± 0.17	0.86 ± 0.08	0.74 ± 0.28	0.84 ± 0.09
4	1.24 ± 0.13	1.54 ± 0.53	1.11 ± 0.16	1.15 ± 0.20
6	1.07 ± 0.21	1.21 ± 0.16	1.56 ± 0.47	1.34 ± 0.24
12	1.36 ± 0.27	0.98 ± 0.03	1.40 ± 0.19	1.30 ± 0.07 *
24	1.25 ± 0.29	1.11 ± 0.08	1.40 ± 0.40	1.23 ± 0.14

Abbreviation: LF: low-fat, HF: high-fat, GTE: green tea extract, LJ: lemon juice. Data were calculated as the percentage to the baseline level. * represents a significant difference (*p* < 0.05) compared to zero hours within the group.
